# Improving colposcopic accuracy for cervical precancer detection: a retrospective multicenter study in China

**DOI:** 10.1186/s12885-022-09498-0

**Published:** 2022-04-10

**Authors:** Bingrui Wei, Bo Zhang, Peng Xue, Samuel Seery, Jiaxu Wang, Qing Li, Yu Jiang, Youlin Qiao

**Affiliations:** 1 Department of Epidemiology and Biostatistics, School of Population Medicine and Public Health, Chinese Academy of Medical Sciences and Peking Union Medical College, Beijing, 100730 China; 2 School of Humanities and Social Sciences, Chinese Academy of Medical Sciences and Peking Union Medical College, Beijing, 100730 China; 3 Faculty of Health and Medicine, Division of Health Research, Lancaster University, Lancaster, LA1 4YW UK; 4 Diagnosis and Treatment for Cervical Lesions Center, Shenzhen Maternity and Child Healthcare Hospital, Shenzhen, 518028 China

**Keywords:** Colposcopy, Accuracy, Cervical cancer, High-grade squamous intraepithelial lesion

## Abstract

**Background:**

Colposcopy alone can result in misidentification of high-grade squamous intraepithelial or worse lesions (HSIL +), especially for women with Type 3 transformation zone (TZ) lesions, where colposcopic assessment is particularly imprecise. This study aimed to improve HSIL + case identification by supplementing referral screening results to colposcopic findings.

**Methods:**

This is an observational multicenter study of 2,417 women, referred to colposcopy after receiving cervical cancer screening results. Logistic regression analysis was conducted under uni- and multivariate models to identify factors which could be used to improve HSIL + case identification. Histological diagnosis was established as the gold standard and is used to assess accuracy, sensitivity, and specificity, as well as to incrementally improve colposcopy.

**Results:**

Multivariate analysis highlighted age, TZ types, referral screening, and colposcopists’ skills as independent factors. Across this sample population, diagnostic accuracies for detecting HSIL + increased from 72.9% (95%CI 71.1–74.7%) for colposcopy alone to 82.1% (95%CI 80.6–83.6%) after supplementing colposcopy with screening results. A significant increase in colposcopic accuracy was observed across all subgroups. Although, the highest increase was observed in women with a TZ3 lesion, and for those diagnosed by junior colposcopists.

**Conclusion:**

It appears possible to supplement colposcopic examinations with screening results to improve HSIL + detection, especially for women with TZ3 lesions. It may also be possible to improve junior colposcopists’ diagnoses although, further psychological research is necessary. We need to understand how levels of uncertainty influence diagnostic decisions and what the concept of “experience” actually is and what it means for colposcopic practice.

## Introduction

Effective cervical cancer screening programs can detect precancers before they progress to invasive cancers [1, 2]; however, there are points in the identification process which are subjective and therefore susceptible to human error. Generally, women with abnormal screening results are referred to colposcopy, and then to biopsy for histologic diagnosis. Colposcopy with biopsy has an important role in determining treatments and further observations. If high-grade squamous intraepithelial or worse lesions i.e., HSIL + is confirmed, treatment is required in most cases [3, 4]. However, colposcopic examination can be inaccurate, since up to 40% of all HSIL + cases are missed in low- and middle-income countries (LMICs) [5, 6]. Of course, colposcopic accuracy can be affected by a number of factors, colposcopists’ skills, screening results i.e., cytology and human papillomavirus [HPV] testing, type of transformation zone (TZ), number of biopsies, etc. [7].

We know that women with lesions in TZ3 are difficult to identify. This is because TZ3 lesions cannot easily be seen with canal-based cytology. Therefore, the number of false negatives related to TZ3 lesions is higher than other types. Poor diagnostic performance is again more evident in LMICs where there is a shortage of skills and experienced colposcopists [8]. A high proportion of women in these countries, between 20–80%, also have TZ3 lesions [9, 10]. Therefore, it is vital we improve colposcopic practice through research and training. Some investigators have reported that adding referral screening results to colposcopic examinations can improve HSIL + detection [11, 12]. Although, colposcopy practitioners do not consider referral screening results with colposcopy, as they might.

A recent study suggested that the use of biomarkers and HPV genotyping could improve diagnostic accuracy for women with TZ3 lesions [13]. However, these findings are based on rather limited data. The study had a small sample size, with approximately only 100 women, and therefore these findings are difficult to generalize, despite being promising. Another issue we face is that there is no comparative data from LMICs which do face a greater burden related to cervical cancer. Therefore, we have a duty to investigate methods for improving HSIL + diagnostics in LMICs such as China, to improve case identification. By supplementing colposcopic diagnosis with referral screening results it is hoped that we can learn to improve HSIL + detection around the world.

## Method

### Study population

Data were retrospectively collected from digital records of women who underwent colposcopic examination from January 2018 to October 2021. All patients had attended one of three colposcopy clinics within different hospitals (municipal and provincial) in mainland China. Women who had previous treatments such as total hysterectomy or history of pelvic radiation, and those who underwent colposcopy but had no histologic report were excluded.

Demographics and clinical data were collected, including age, cytological results, HPV status, TZ types, colposcopic diagnosis, colposcopists’ skills and histological results. This study was conducted in accordance with the Declaration of Helsinki and received ethical approval from the institutional review board (IRB) of Chinese Academy of Medical Sciences and Peking Union Medical College (CAMS/PUMC). The need for informed consent was waived since the study was retrospective and personal information was anonymized.

### Cytology and HPV testing

Cytology results were classified into five categories according to the Bethesda System [14] including, no intraepithelial lesion cells and malignant cells (NILM), atypical squamous cells of unknown significance (ASC-US), low-grade squamous intraepithelial lesion (LSIL), atypical squamous cells of cannot exclude high-grade squamous intraepithelial lesion (ASC-H), high-grade squamous intraepithelial lesion and/or squamous cervical carcinoma (HSIL +). The type of HPV test used was not recorded in this instance because it was not considered pertinent to this study. High-risk HPV (hr-HPV) types were defined as either HPV16/18, non-16/18 hrHPV or hrHPV negative.

### Colposcopy and histology diagnosis

Referral to colposcopic examination was based on the 2011 International Federation of Cervical Pathology and Colposcopy (IFCPC) [15]. All colposcopies were performed by different colposcopists (junior and senior), which were divided into two groups: those with more than 5-year experience, and those with less than 5-year experience using digital colposcopes.

General assessment was conducted to check cervical visualizations, which included visible TZ1/2 and not visible TZ3. All colposcopically detected abnormalities were directly biopsied. If necessary, an endocervical curettage was performed after cervical biopsies. The colposcopic diagnosis was described as normal/benign findings, low-grade, high-grade.

Histological diagnosis was performed by experienced histologists from local hospitals with disagreements considered in consultation with histologists’ consultations. Results were classified as normal, low grade squamous intraepithelial lesion (LSIL), and high-grade squamous intraepithelial lesion or worse (HSIL +) according to a revised version standard of World Health Organization (WHO) classifications [16]. The histologic results were taken as the gold standard. When analyzing biopsies, excision specimens and/or endocervical curettage together, the final histological diagnosis was considered the worst grade of dysplasia present.

### Statistical analysis

Data analysis was performed using SPSS (version 24.0) and R (version 4.0.5). Accuracy, sensitivity, and specificity were used to assess diagnostic performance for HSIL + . Findings were compared using a standard Chi-square test.

Univariate and multivariate logistic regression analyses with an enter approach were performed to assess independent factors which are reported as odds ratios (OR) with 95% CI for detecting HSIL + . All* p* values presented are two-sided and differences were considered statistically significant with *p* values lower than 0.05.

## Results

### Clinical characteristics of study population

Data from 2,417 women were enrolled into this analysis. Table 1 provides summaries of clinical characteristics of colposcopy population including age, cytology results, HPV status, the types of TZ, colposcopy diagnosis and histology results. The women were aged 19–78 and the largest age group (67.7%) was aged 30–45 years. Almost 56% women is TZ3. The most common cytology result, reflecting referral cytology, was NILM (53.2%), ASC-US (20.2%), LSIL (12.9%), followed by ASC-H (6.1%) and HSIL + (7.6%), while the most common HPV type category was negative (36.2%), non-16/18 hrHPV positive (33.6%), and HPV16/18 positive (30.2%). The colposcopy diagnosis categories in order of severity were normal/benign (31.9%), low-grade (22.9%) and high-grade (45.2%). Histology showed HSIL + in 44.8% of cases.

**Table 1:** Clinical characteristics of study population (*N* = 2417)

Characteristics	N	(%)
**Total**	2417	100
**Age**		
< 30	325	13.4
30–35	823	34.1
36–45	812	33.6
> 45	457	18.9
**Cytology**		
NILM	1287	53.2
ASC-US	488	20.2
LSIL	312	12.9
ASC-H	148	6.1
HSIL +	182	7.6
**HPV status**		
Negative	874	36.2
Non-16/18 hrHPV positive	811	33.6
HPV16/18 positive	732	30.2
**Transformation zone**		
TZ1/2	1061	43.9
TZ3	1356	56.1
**Colposcopy diagnosis**		
Normal/benign	771	31.9
Low grade	554	22.9
High grade	1092	45.2
**Histology**		
Normal	654	27.1
LSIL	680	28.1
HSIL +	1083	44.8
**Colposcopist’s skills**		
Junior	1537	63.6
Senior	880	36.4

Abbreviations: *NILM* no intraepithelial lesion cells and malignant cells, *ASC-US* atypical squamous cells of unknown significance, *LSIL* low-grade squamous intraepithelial lesion, *ASC-H* atypical squamous cells of cannot exclude high-grade squamous intraepithelial lesion, *HSIL* + high-grade squamous intraepithelial lesions or worse, *hrHPV* high-risk human papillomavirus, *TZ* transformation zone

### Evaluation of the diagnostic performance of colposcopy for detecting HSIL + 

The overall colposcopic performance and its subgroup performance in different TZ types and colposcopist’s skills for detecting HSIL + are showed in Table 2. The overall diagnostic accuracy, sensitivity and specificity were 72.9%, 70.2%, and 75.1%, respectively. For women with different TZ types detecting HSIL + , the accuracy (75.4% versus 70.9%, *p* = 0.014) and sensitivity (77.9% versus 62.2%, *p* < 0.001) of TZ1/2 were significantly higher than those with TZ3, while there was no statistical difference for specificity (72.7% versus 76.6%, *p* = 0.109). For women diagnosed by different colposcopists’ skills detecting HSIL + , the accuracy (69.6% versus 78.6%, *p* < 0.001) and sensitivity (62.0% versus 79.8%, *p* < 0.001) of senior colposcopists were significantly higher than those with the junior colposcopists. As for specificity (77.1% versus 74.3%, *p* = 0.290), the senior colposcopists was slightly higher than junior colposcopists, but no statistical difference between different colposcopists’ skills.

**Table 2:** Diagnostic performance of colposcopy for detecting HSIL +

	**Accuracy, % (95%CI)**	**Sensitivity, % (95%CI)**	**Specificity, % (95%CI)**
**Colposcopy**	72.9 (71.1–74.7)	70.2 (67.5–72.9)	75.1 (72.8–77.4)
**TZ types**			
TZ1/2	75.4 (72.8–78.0)	77.9 (74.4–81.4)	72.7 (68.8–76.6)
TZ3	70.9 (68.5–73.3)	62.2 (58.1–66.3)	76.6 (73.7–79.5)
**Colposcopist’s skills**			
Junior	69.6 (67.3–71.9)	62.0 (58.1–65.9)	74.3 (71.5–77.1)
Senior	78.6 (75.9–81.3)	79.8 (76.3–83.3)	77.1 (72.9–81.3)
**TZ types and colposcopist’s skills**			
Junior and TZ1/2	70.8 (67.4–74.2)	71.7 (66.5–76.9)	70.0 (65.4–74.6)
Junior and TZ3	68.7 (65.6–71.8)	52.5 (46.8–58.2)	77.3 (73.8–80.8)
Senior and TZ1/2	83.6 (79.9–87.3)	84.9 (80.5–89.3)	81.0 (74.2–87.8)
Senior and TZ3	74.8 (71.0–78.6)	74.4 (68.9–79.9)	75.2 (69.9–80.5)

Abbreviations: *CI* confidence interval, *HSIL* + high-grade squamous intraepithelial lesions or worse, *TZ* transformation zone

### Clinical factors analysis affecting the colposcopic accuracy of detecting HSIL + 

Table 3 presents univariate and multivariate logistic regression analysis. We showed that the age group, cytology and, HPV status, TZ types, and colopscopists’ skills appear as clinical influences over colposcopic accuracy of detecting HSIL + . In multivariate logistic regression, women whose HPV status were HPV16/18 (OR, 4.45), and cytological results were LSIL (OR, 2.42), ASC-H (OR, 3.41) and HSIL + (OR, 28.26) appears to positively correlate with higher odds of accurate detecting histological HSIL + . These findings indicate that a combination of the referral screening test results, as this could improve the colposcopic accuracy.

**Table 3:** Logistic regression analysis of factors affecting colposcopic accuracy for detecting HSIL +

Factors	Univariate		Multivariate	
	**OR (95%CI)**	***p* value**	**OR (95%CI)**	***p* value**
**Age**				
< 30	1.00		1.00	
30–35	0.75 (0.49–1.17)	0.210	0.63 (0.38–1.03)	0.066
36–45	0.86 (0.56–1.33)	0.505	1.23 (0.75–2.03)	0.420
> 45	0.58 (0.37–0.91)	0.019	0.37 (0.21–0.64)	< 0.001
**Cytology**				
NILM	1.00		1.00	
ASC-US	1.27 (0.91–1.76)	0.158	2.11 (1.42–3.15)	< 0.001
LSIL	1.76 (1.21–2.54)	0.003	2.42 (1.56–3.70)	< 0.001
ASC-H	2.59 (1.52–4.41)	< 0.001	3.41 (1.89–6.17)	< 0.001
HSIL +	12.48 (5.91–26.34)	< 0.001	28.26 (12.38–64.52)	< 0.001
**HPV status**				
Negative	1.00		1.00	
Non-16/18 hrHPV positive	1.58 (1.11–2.25)	0.011	1.50 (1.01–2.23)	0.045
HPV16/18 positive	3.95 (2.69–5.80)	< 0.001	4.45 (2.87–6.90)	< 0.001
**Transformation zone**				
TZ1/2	1.00		1.00	
TZ3	0.47 (0.36–0.61)	< 0.001	0.45 (0.34–0.62)	< 0.001
**Colposcopist’s skills**				
Junior	1.00		1.00	
Senior	2.43 (1.84–3.20)	< 0.001	2.24 (1.63–3.07)	< 0.001

Abbreviations: *OR* odds ratio, *CI* confidence interval, *NILM* no intraepithelial lesion cells and malignant cells, *ASC-US* atypical squamous cells of unknown significance, *LSIL* low-grade squamous intraepithelial lesion, *ASC-H* atypical squamous cells of cannot exclude high-grade squamous intraepithelial lesion, *HSIL* + high-grade squamous intraepithelial lesions or worse, *hrHPV* high-risk human papillomavirus, *TZ* transformation zone

### Diagnostic performance of colposcopy combined with referral screening results for detecting HSIL + 

**Fig. 1:**
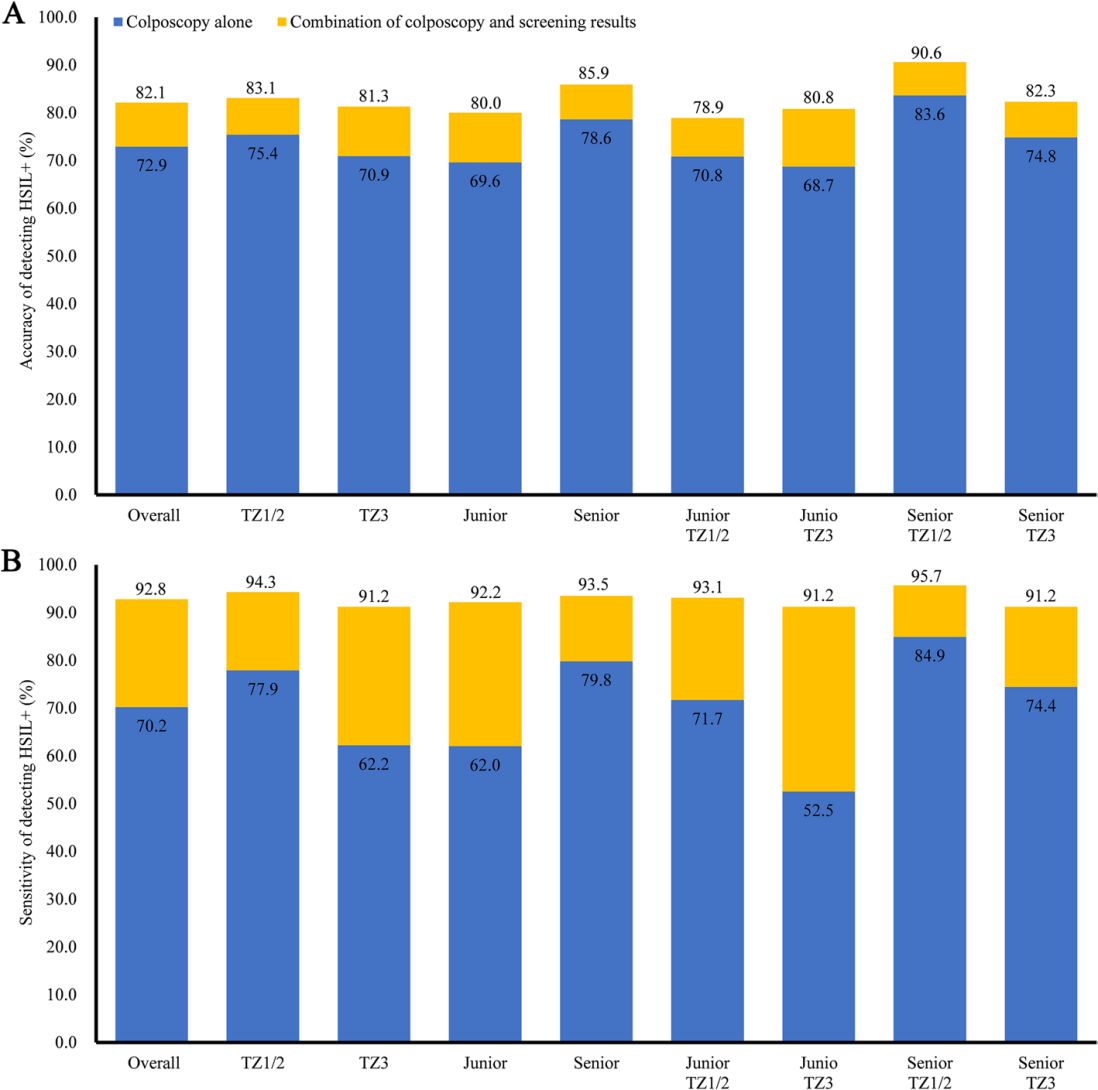
**A** Increased yields of colposcopic accuracy by supplementing screening results for detecting HSIL + . **B** Increased yields of colposcopic sensitivity by supplementing screening results for detecting HSIL + . Notes: Blue bar, diagnostic performance of colposcopy alone in detecting HSIL + ; Yellow bar, increased yields of colposcopic performance by supplementing screening results, Overall, all colposcopy population; TZ1/2, type 1 or type 2 transformation zone; TZ3, type 3 transformation zone; Junior: juior colposcopist; Senior: senior colposcopist

Table 4 reports the diagnostic performance of adding the referral screening test results to colposcopy for detecting HSIL + . Fig. 1 shows the incremental accuracy and sensitivity for HSIL + of adding the referral screening test results. The overall colposcopic accuracy and sensitivity were improved to 82.1% and 92.8%, respectively by adding the referral screening test results. Despite slight decline for specificity observed, there is no statistical significance (for overall, *p* = 0.33; for subgroups, all *p* > 0.05). Similar increments and were observed in all subgroups analyses. Among women with a TZ3, the accuracy and sensitivity of yields for HSIL + increased from 70.9% to 81.3%, and from 62.2% to 91.2%, respectively, and among women diagnosed by junior colposcopists, the corresponding yields for HSIL + increased from 69.6% to 80.0%, and from 62.0% to 92.2%, respectively. we found that the highest accuracy (12.1%) and sensitivity (38.7%) of in increment yields for HSIL + were observed in the subgroup of women with a TZ3, and diagnosed by junior colposcopists.

**Table 4:** Diagnostic performance of colposcopy combined with referral screening test results for detecting HSIL +

	**Accuracy, % (95%CI)**	**Sensitivity, % (95%CI)**	**Specificity, % (95%CI)**
**Colposcopy**	82.1 (80.6–83.6)	92.8 (91.3–94.3)	73.5 (71.1–75.9)
**TZ types**			
TZ1/2	83.1 (80.8–85.4)	94.3 (92.4–96.2)	71.2 (67.3–75.1)
TZ3	81.3 (79.2–83.4)	91.2 (88.8–93.6)	74.9 (71.9–77.9)
**Colposcopist’s skills**			
Junior	80.0 (78.0–82.0)	92.2 (90.0–94.4)	72.4 (69.6–75.2)
Senior	85.9 (83.6–88.2)	93.5 (91.3–95.7)	76.0 (71.7–80.3)
**TZ types and colposcopist’s skills**			
Junior and TZ1/2	78.9 (75.8–82.0)	93.1 (90.2–96.0)	68.2 (63.6–72.8)
Junior and TZ3	80.8 (78.2–83.4)	91.2 (88.0–94.4)	75.3 (71.7–78.9)
Senior and TZ1/2	90.6 (87.7–93.5)	95.7 (93.2–98.2)	80.2 (73.2–87.2)
Senior and TZ3	82.3 (78.9–85.7)	91.2 (87.6–94.8)	74.0 (68.6–79.4)

Additional description: Compared with single colposcopic results, the integration of colposcopic results with abnormal screening results (i.e., HPV and/or cytology) is more likely to identify HSIL + cases.

Abbreviations: *CI* confidence interval, *HSIL* +  High-grade squamous intraepithelial lesions or worse, *TZ* Transformation zone

## Discussion

The aim of this study was to supplement colposcopic diagnosis with referral screening results with a view to improving HSIL + detection in China, and around the world. Data from 2,417 women who attended colposcopy clinics across China were collated which enabled us to assess colposcopic performance. Evidence was then stratified according to TZ types and around colposcopists’ skills and experience. The primary goal was determined whether supplementing colposcopic diagnosis with referral screening results can add clinical value. Overall, colposcopy alone had a relatively low level of accuracy i.e., 72.9% for detecting HSIL + however, this was not surprising. Colposcopic agreement with biopsies varies substantially between and within countries but also between specialist clinics. This is because a colposcope is operated by human clinicians over a period of 20–40 min, which increases the risk of error.

The sensitivity of colposcopy alone for detecting HSIL + in this study was 70.2%, with a specificity of 75.1%. These appear comparable to previous studies conducted in China. For example, in a previous study we found that colposcopic accuracy for detecting HSIL + was 69.7% [17]. Likewise, Li et al. reported 64.95% accuracy for colposcopically directed biopsy when identifying HSIL + cases [18]. Another study by Fan et al. [19] found agreement between colposcopic diagnosis and histology of 65.5% in China. Taken together this evidence suggests that colposcopy is a useful tool to detect HSIL + but it is imprecise. As has been mentioned, colposcopic accuracy is biased on colposcopists’s level subjective experience, TZ and etc. Each of these cause inaccuracies which have prompted studies to improve colposcopic HSIL + detection. It is important to be aware that these inaccuracies have real-world consequences which should be avoided, if at all possible. Many may simply defer to biopsy but we must also consider the psychological implications for patients and the economics which all health systems must factor into treatment pathways.

One clear issue that we must address is how colposcopists’ skills can affect colposcopic accuracy for detecting HSIL + . Therefore, in this study we performed subgroup analysis of senior and junior colposcopists. Unsurprisingly, we found colposcopic accuracy was higher for senior colposcopists with more than five years of experience. This is consistent with other findings from around the world. For example, Baum et al. reported related evidence that junior colposcopists have a tendency to overestimate [20] however, some have found opposing evidence. For instance, Stuebs et al. [11] found no significant difference between colposcopists’ according around experience. Although, their evidence may be biased because of patient selection. Patients with more complex symptoms are also more likely to be attended to by senior colposcopits. So, it is fair to say that experience is more likely to benefit patients but can also create more assumptions or lapses which lead to misdiagnoses.

Transformation zones are the most important anatomical entities where CIN and invasive carcinoma arise. However, as we know colposcopy is not always adequately performed. Among other reasons, this is because transformation zones are often obscured from view but inflammation, atrophy, bleeding etc. This is especially true for TZ3 lesions which can be ‘tucked-inside’ the cervix and therefore are less visible. We found that 56.1% of all cases had TZ3 lesions, which likely meant that colposcopies were unsatisfactorily completed. This seems high but again this varies substantially. For example, Zhang et al. [10]. studied 1,838 cases and found 18.2% were TZ 1 or 2, and the remaining 81.8% were TZ3 cases. While Fan et al. [19] found 83.6% were TZ1 or 2, and 16.4% were TZ3 of the 1,892 cases studied. In a German study researchers found that TZ3 lesions accounted for 81% of the 3,761 cases studied [21]. Again, these variations may be explained by the distribution of lesions within the cervix. We found significant heterogeneity in the distribution of different TZs in different studies although, this would need to be systematically reviewed. It may be possible to develop a type of transpositional image-based meta-analysis to study transformation zones although it is beyond this study to discuss this further.

Evidence from this study suggests there is a higher accuracy (75.4% versus 70.9%) and sensitivity for HSIL + (77.9% versus 62.2%) in women with TZ1/2 compared to women with TZ3. However, there was no significant difference in TZ types for specificity (72.7% versus 76.6%). Stuebs et al. [11]. reported similar results with higher accuracies for TZ1/2 and lower accuracies for TZ3. In another study, it has also been found that the accuracy of TZ1 or 2 is higher than for TZ3 [22]. These studies suggest that transformation zones around of critical importance, and affect colposcopic accuracy, especially for TZ3. This is also supported by meta-analytical results by Ren et al. [7]. However, the key issue is how aware a colposcopist is of the unsatisfactory nature of the examination and of course, what she or he decides to do next for the patient. Further research is needed into the decision-making process and psychological factors which drive “next-step” processes in the diagnostic pathway. We need insight into levels of uncertainty which initiate colposcopists to order biopsies or to schedule follow-up examinations. Knowing this would take us beyond the notion that ‘experience’ as the important factor, and ground us into developing more sophisticated colposcopy training.

We performed subgroup analysis according to TZ types and colposcopists’ levels of experience. In the junior colposcopist subgroup, we found similar results, with a higher sensitivity of 71.7% for TZ1/2, compared to TZ3 which was 52.5%. For senior colposcopists, we found that the difference of colposcopic sensitivity between women with TZ1/2 and TZ3 was not so largely. One explanation for this is that, colposcopists with more than five years of clinical experience are more likely to accurately identify TZ3 HSIL + . Although, it may also mean that a more senior colposcopist is more likely to seeking confirmation and rather use biopsy to disprove a diagnostic assertion. It is therefore, important that the 2011 IFCPC guidelines have incorporated classification transformation zone classification as an obligatory terminology [15]. However again, there are discrepancies due to the lack of prospective and randomized clinical trials which record and report this data. Further research is needed to understand the dynamics involved and to standardize guidelines because at present transformation zone terminology is not considered in the ASCCP guidelines [23, 24].

We also conducted univariate and multivariate analysis to explore factors which influence colposcopic accuracy at detecting HSIL + . We found that the age, cytology, HPV test results, TZ type and the colposcopists’ skills are all independent factors. Among influential factors, cytology and HPV testing appear to be significantly related to HSIL + detection with relatively high odds ratios. These findings are also consistent with previous clinical studies conducted in China [12, 17, 25]. This is understandable because women referred to colposcopy with high-risk screening results (e.g., HPV16/18 + and high-grade cytology) are more likely to be HSIL + cases. By contrast, women with low-risk screening results (e.g., HPV16/18- and ASC-US cytology) were at low-risk of HSIL + . Therefore, European and American relevant guidelines highlight the value of adding screening results to colposcopy impression for improved HSIL + case identification [24, 26].

In order to develop this body of evidence, we supplemented colposcopic diagnosis with referral screening results. By adding this we managed to increase accuracy by 9.2%, and sensitivity by 22.6%, which is considerable and despite slightly lowering specificity by 1.6%. We also observed similar results in different TZ types and according to colposcopists’ skills. Although, the greatest sensitivity increase was observed for previously poor performance in the TZ3 subgroup and in junior colposcopists. Overall, our results extend the evidence from clinical studies which suggest that combining colposcopic findings with referral screening results can markedly improve HSIL + detection.

However, it is important consider what else could be done to further improve colposcopic accuracy and ensure the WHO’s goal of eliminating cervical cancer worldwide by 2030 can be achieved. Of course, HPV vaccinations will surely help as will the move from operator-dependent cytology to less operator-dependent HPV detection. Although, the practice of colposcopy faces a number of unpredictable challenges [27]. Therefore, guidelines should be regularly updated (according to best evidence) to meet the needs of real-world clinical practice. A number of possible factors (e.g., pre-colposcopy assessment, extended HPV genotyping, biomarkers, dual staining and methylation, etc.) could potentially improve diagnostic performance although, these are not yet available as point-of-care testing. Recently, the Polish Society of Colposcopy and Cervical Pathophysiology has suggested to include a series of pre-colposcopy assessments, detection techniques and HPV vaccination status, to optimize colposcopy practice toward tailored management [28]. Our findings indicate the benefits of adding screening results to colposcopic diagnosis to better identify HSIL cases. However, more data should be provided to support optimal colposcopy strategy development, based on supplementary measures which improve the diagnostic accuracy of colposcopy. Unfortunately, as well as trying to manage diagnostic uncertainty and our patients’ well-being, we are also increasingly being encouraged to consider the financial implications of prognostics. Moreover, there is a shortage of experienced colposcopists, especially in LMICs. Perhaps, artificial intelligence-guided colposcopy can improve this form of diagnostics [8, 29] although we need to test and validate new models. As always, these advances will have implications for medical education which should not be left as an afterthought.

To the best of our knowledge, this is the first study to consider the diagnostic value for identifying HSIL + in women with different TZs and colposcopist’ skills by integrating colposcopic impressions with referral screening results across a Chinese multicenter study. However, there were several limitations that should be mentioned. Firstly, this was a retrospective study design which comes with a number of well-reported issues. Second, we previously found that taking multiple colposcopy-directed biopsies can increase HSIL detection [30–32], therefore we did not extract all pertinent data for analysis. Third, we tried to investigate the influence age, although this was not included because we were unable to extract sufficient data to understand how age influences HSIL + detection. We are planning further research to understand factors involved in women’s development which impact on case detection. Finally, we have only studied colposcopic accuracy for detecting HSIL + . The data required to discern differences between HSIL + , cervical intraepithelial neoplasia grade 2 or worse, and CIN3 + are still needed.

## Conclusion

Our findings reaffirm that colposcopic HSIL + detection is imprecise, especially for women with TZ3 lesions and those diagnosed by junior colposcopists. It is also possible to improve upon this by supplementing colposcopic findings with referral screening results. Perhaps, we could develop a type of transpositional image-based meta-analysis to study TZs although this is interdisciplinary research which requires more thought. We also need to conduct more psychological research to gain insight into colposcopists’ levels of uncertainty and how they acknowledge and respond to this sensation. At present, the concept of “experience” is based solely upon a time threshold, we have no understanding of what “experience” actually means for colposcopists.

## Data Availability

The datasets generated and/or analysed during the current study are not publicly available due personal information protection, patient privacy regulation, and medical institutional data regulatory policies, etc., but are available from the corresponding author on reasonable request and with permission of the Chinese Academy of Medical Sciences and Peking Union Medical College data sharing committee.

## Abbreviations

ASC-US: Atypical squamous cells of unknown significance
ASC-H: Atypical Squamous Cells of cannot exclude High-grade squamous intraepithelial lesion
CAMS/PUMC: Chinese Academy of Medical Sciences and Peking Union Medical College
HSIL +: High-grade Squamous Intraepithelial or worse lesions
HPV: Human Papillomavirus
hrHPV: High-risk HPV
IRB: Institutional Review Board
IFCPC: International Federation of Cervical Pathology and Colposcopy
LMICs: Low- and Middle-Income Countries
LSIL: Low-grade Squamous Intraepithelial Lesion
NILM: No Intraepithelial Lesion cells and Malignant cells
OR: Odds Ratios
TZ: Transformation Zone
WHO: World Health Organization
